# Antioxidants inhibit cell senescence and preserve stemness of adipose tissue-derived stem cells by reducing ROS generation during long-term in vitro expansion

**DOI:** 10.1186/s13287-019-1404-9

**Published:** 2019-10-17

**Authors:** Naishun Liao, Yingjun Shi, Cuilin Zhang, Youshi Zheng, Yingchao Wang, Bixing Zhao, Yongyi Zeng, Xiaolong Liu, Jingfeng Liu

**Affiliations:** 1 The United Innovation of Mengchao Hepatobiliary Technology Key Laboratory of Fujian Province, Mengchao Hepatobiliary Hospital of Fujian Medical University, Fuzhou, 350025 People’s Republic of China; 2 Liver Disease Center, The First Affiliated Hospital of Fujian Medical University, Fuzhou, 350007 People’s Republic of China; 3 Mengchao Med-X Center, Fuzhou University, Fuzhou, 350116 People’s Republic of China; 4 The Liver Center of Fujian Province, Fujian Medical University, Fuzhou, 350025 People’s Republic of China

**Keywords:** Adipose tissue-derived mesenchymal stem cells, Reduced glutathione, Melatonin, ROS, Senescence, Stemness

## Abstract

**Background:**

Adipose tissue-derived mesenchymal stem cells (ADSCs) are promising candidates for regenerative medicine. However, long-term in vitro passaging leads to stemness loss and cell senescence of ADSCs, resulting in failure of ADSC-based therapy.

**Methods:**

In this study, ADSCs were treated with low dose of antioxidants (reduced glutathione and melatonin) with anti-aging and stem cell protection properties in the in vitro passaging, and the cell functions including stem cell senescence, cell migration, cell multidirectional differentiation potential, and ROS content were carefully analyzed.

**Results:**

We found that GSH and melatonin could maintain ADSC cell functions through reducing cell senescence and promoting cell migration, as well as by preserving stemness and multidirectional differentiation potential, through inhibiting ROS generation during long-term expansion of ADSCs.

**Conclusions:**

Our results suggested that antioxidant treatment could efficiently prevent the dysfunction and preserve cell functions of ADSCs after long-term passaging, providing a practical strategy to facilitate ADSC-based therapy.

## Background

Adipose tissue-derived mesenchymal stem cells (ADSCs) are candidates for treating various diseases in the fields of regenerative medicine, because of their multidirectional differentiation potential, immunoregulation, and potent paracrine functions [1, 2]. Until 2018, more than 190 clinical trials of ADSC-based therapy are described in the database of “ClinicalTrials.gov,” and most clinical applications need to implement 10–100 million ADSCs for each therapy [3]. Given the fact that the number of primitive stem cells in adipose tissues cannot meet the actual needs, in vitro expansion is an indispensable procedure to obtain a sufficient number of cells for their clinical applications. However, ADSCs are easy to lose their primitive stemness and exhibit a reduced proliferation potential and cell senescence during long-term in vitro expansion [4–6]. Herein, the quantity and quality of ADSCs expanded in vitro severely affect their clinical efficacy. For obtaining the optimized ADSC therapy outcomes, maintaining the cell functions and stemness as well as reducing cell senescence are crucial for the in vitro expansion of ADSCs.

Cell functions of ADSCs are primitively retained by the in vivo physiological microenvironment, including cell-cell and cell-matrix interactions [7]. Currently, many strategies such as hypoxia [8, 9] and three dimensional (3D) culture methods [4, 10] have been used to maintain ADSC functions by providing proper niche or mimicking native microenvironment. However, these strategies will bring some disadvantages. For instance, hypoxia was previously reported to induce genomic instability for in vitro-expanded ADSCs [11] although it could maintain stem cell stemness via delaying cell proliferation and arresting cell cycle [12, 13]. Furthermore, it is difficult to ensure the source and quality stability of 3D culture materials for clinical purpose in ADSC expansion [6]. Therefore, it is still urgent to find a more applicable method to maintain ADSC functions during in vitro expansion.

Accumulating evidence has suggested that redox homeostasis plays a central role in maintaining stemness and reducing stem cell senescence [14–16]. In particular, disturbance stem cell redox homeostasis by excessive production of reactive oxygen species (ROS) could lead to oxidative stress, resulting in stem cell dysfunctions such as stem cell senescence and lost stemness during long-term in vitro expansion [14, 17]. Moreover, it has been shown that ADSCs also undergo accumulation of ROS in large scale in vitro expansion [18], and precise regulation of ROS is crucial for cellular homeostasis of ADSCs [19]. Under physiological conditions, stem cells maintain low levels of ROS to preserve their stemness and to remain quiescent in mammals [14, 15, 20]. Thus, it is necessary to reduce excessive ROS production during long-term in vitro expansion of ADSCs.

Cellular redox homeostasis is maintained by antioxidant systems including enzymes and antioxidant molecules to scavenge excessive ROS production [21]. Among them, glutathione is one the most important antioxidant molecule that plays a critical role in cellular ROS neutralization [22]. Interestingly, it has been proved that a high level of glutathione, which protects against the unfavorable DNA damage [23], is a prerequisite for maintaining stem cell functions during in vitro expansion [21]. Considering that exogenous glutathione could improve intracellular glutathione synthesis via the γ-glutamyl cycle [24], adding exogenous glutathione would be a benefit for preserving cell functions of ADSCs during in vitro expansion.

Melatonin (*N*-acetyl-5-methoxytryptamine), a molecule produced by the pineal gland, is an important sleep hormone in the circadian rhythm of the organism [25]. More commonly known as the sleep hormone, melatonin also has many other crucial properties, including antioxidant, anti-inflammatory, and antiapoptotic effects [25, 26]. Emerging evidences suggested that melatonin could be used to maintain stemness of bone marrow-derived mesenchymal stem cells (BMSCs) during in vitro expansion [6, 27–29]. Melatonin treatment could be also used to promote osteogenic differentiation of ADSCs by enhancing alkaline phosphatase activity [30]. Meanwhile, Han and colleagues showed that melatonin treatment promotes cell survival of ADSCs in vivo, resulting facilitated ADSC therapy for myocardial infarction [31]. Furthermore, Yun and colleagues showed that melatonin could rescue uremic toxin *p-Cresol*-induced MSC senescence [32]. However, the effects of melatonin on cell senescence of ADSCs and on the maintenance of stemness have not yet been fully investigated.

In this study, antioxidants including reduced glutathione (GSH) and melatonin were used to prevent stemness loss and reduction of cell senescence in long-term subculture of ADSCs. We found that GSH and melatonin could preserve stemness and reduce cell senescence of ADSCs by reducing ROS generation during long-term in vitro expansion. Hence, this study may provide a novel small molecule-based approach for maintaining cell functions of ADSCs during in vitro culture.

## Methods

### ADSC isolation and culture

Isolation of ADSCs was performed according to our previous descriptions [33, 34]. Briefly, adipose tissues were collected from subcutaneous inguinal area of male C57BL/6 mice (4 weeks old; *n* = 10) and then cut into small pieces (about 0.1 mm^3^ size) and digested with 0.1% collagenase (type I; Sigma-Aldrich, USA) in HBSS containing calcium and magnesium (Hyclone, USA) at 37 °C for 60 min. Then, digestive solutions were subsequently neutralized by α-MEM (Hyclone, USA) containing 20% FBS (Gibco, USA). Afterwards, the collected solutions were filtered through a cell strainer (100 μm) to eliminate the red blood cells with osmotic lysates (Biyuntian Biological Co., Ltd., Shanghai, China). Finally, the cells were collected and seeded at a density of 1 × 10^6^/mL on T-75 flasks with α-MEM containing 10% FBS and 1% penicillin/streptomycin (Gibco, USA). Once the cells reached confluence, they were enzymatically detached using 0.25% trypsin-EDTA solution (Gibco, USA) and passaged at a ratio of 1:3.

### Antioxidant treatment

Antioxidants including GSH and melatonin were obtained from Aladdin Chemical (Shanghai, China). Each antioxidant was firstly dissolved in DMSO (Sigma-Aldrich, USA) to the concentration of 1 mM. For antioxidant treatment, the ADSCs were cultured with complete medium (α-MEM containing 10% FBS and 1% penicillin/streptomycin) supplemented with 10 μM antioxidant since the first passage. The antioxidant-treated medium was changed every 3 days. All cells were rinsed with PBS three times to remove any residual antioxidant before further analysis.

### Cell proliferation assay

After treating the ADSCs at passages 3, 6, and 9 with GSH, melatonin, or the combination of GSH and melatonin, the cells were seeded at a density of 1 × 10^4^/well in a 96-well plate and incubated at 37 °C with 5% CO_2_. After incubation for 24 h, 48 h, or 96 h, cell viability was analyzed by using a CCK-8 cell proliferation kit following the manufacture’s protocol (TransGen, Beijing, China). A microplate reader (Spectra Max M5) was used to measure the absorbance of the solution in each well at 450 nm.

### Senescence-associated β-galactosidase (SA-β-gal) staining

To determine the effects of GSH and melatonin on cell senescence of ADSCs, SA-β-gal staining was used. Briefly, after treating ADSCs with GSH and melatonin for passages 3, 6, and 9, the cells were seeded at a density of 5 × 10^4^/well in a 6-well plate with complete medium and incubated for 24 h at 37 °C in 5% CO_2_. Afterwards, the cells were fixed with 4% paraformaldehyde (PFA, Sigma-Aldrich, USA) at room temperature for 20 min and subsequently stained by SA-β-gal staining kit (Beyotime, Shanghai, China) following the manufacturer’s instructions. Positive senescent cells stained in blue were observed using an inverted microscope (Zeiss, Germany).

### Cell migration assay

To explore the effects of GSH and melatonin on the migration of ADSCs, a scratch wound healing assay and the Transwell migration assay were used. For the scratch wound healing assay, the migration ability of ADSCs was evaluated by means of a wound healing assay using a 35-mm culture insert dish (Ibidi, Germany) according to the manufacture’ introduction; 24 h later, the plugin was removed to leave a cell-free gap (about 500 μm); finally, the migration rate into this “wound area” was observed and measured using a Carl Zeiss microscope (Zeiss, Germany). For the Transwell migration assay, 5 × 10^5^ cells were loaded into the upper compartment of the Transwell plate (pore size, 8 μm; Millipore, USA) with α-MEM containing 2% FBS, and the lower compartment of the chamber was filled with complete medium; after culturing for 24 h, the filters were fixed with 4% PFA and stained with crystal violet; afterwards, the cells on the upper surface of the Transwell plates were removed using a cotton swab, and the lower surface of the filter was observed using an inverted microscope (Zeiss, Germany); to further quantify the migration rate of ADSCs, we randomly selected five fields (at a magnification of × 200) to count the number of migrated cells.

### Multi-differentiation potential analysis

To assess the effects of antioxidants on the multilineage differentiation potential ability, the ADSCs were cultured in osteogenic, adipogenic, and chondrogenic induction media as previously described [35]. For osteogenic differentiation, the ADSCs were cultured at a density of 5 × 10^4^/well in a 24-well plate and treated with complete medium supplied with 0.1 mM dexamethasone (Sigma-Aldrich, USA), 50 mM ascorbate-2-phosphate (Sigma-Aldrich, USA), and 10 mM β-glycerol phosphate (Sigma-Aldrich, USA) for 4 weeks; afterwards, the cells were fixed with 4% PFA and stained with 0.1% Alizarin Red S solution (Solarbio, Beijing, China). For adipogenic differentiation, the ADSCs were cultured at a density of 5 × 10^4^/well in a 24-well plate; treated with complete medium supplied with 10 mM insulin (Sigma-Aldrich, USA), 1 mM dexamethasone, 0.5 mM isobutyl-methylxanthine (Sigma-Aldrich, USA), and 200 mM indomethacin (Sigma-Aldrich, USA) for 2 weeks; and finally stained with an Oil Red O staining kit (Nanjing Jiancheng Bioengineering Research Institute, Nanjing, China) following the manufacturer’s protocol. For chondrogenic differentiation, the cells were cultured at a density of 5 × 10^4^/well in a 24-well plate and treated with α-MEM containing 1% FBS, 10 ng/mL TGF-β1 (PeproTech, USA), 6.25 mg/mL insulin, and 50 nM ascorbate-2-phosphate for 4 weeks; finally, the cells were stained with an Alcian blue staining kit (Nanjing Jiancheng Bioengineering Research Institute, Nanjing, China) according to the manufacture’s protocol.

### ROS content assay

The intracellular ROS levels were analyzed by ROS assay kit (Beyotime, Shanghai, China) according to the manufacture’s introduction. Briefly, the cells were cultured at a density of 1 × 10^5^/well in the 35-mm-diameter confocal dishes. After 24 h, the cells were incubated with 25 mM 2′, 7′-dichlorofluorescein diacetate (DCFH-DA) at 37 °C for 30 min. Afterwards, the ROS content of ADSCs was analyzed by LSM780 confocal microscope (Zeiss, Germany) at 488-nm excitation and 525-nm emission wavelengths. To further quantify the results, we randomly selected five fields (at a magnification of × 400) to calculate the fluorescence intensity of each condition. The fluorescence intensity of DCFH-DA was quantified in the “Histo” module of the ZEN 2012 Light Edition imaging analysis system (Zeiss, Germany).

### Superoxide and NOXs content assay

To further determine the antioxidative effect of GSH and melatonin on ADSCs, different passages of ADSCs were collected, and the content of NOXs was measured using a NOXs test Kit (Nanjing Jiancheng Bioengineering Research Institute, Nanjing, China) following the manufacture’s protocol. Finally, the content of NOXs was normalized to the corresponding protein concentration of ADSCs, which was analyzed by a bicinchoninic acid (BCA) assay kit (TransGen Biotech, Beijing, China). To further determine the content of superoxide in ADSCs, a Superoxide Assay Kit (Beyotime Institute of Biotechnology, Shanghai, China) was used. Briefly, the ADSCs were plated at a density of 5 × 10^3^ cells/well in 96-well plates. Twenty-four hours later, the cell culture supernatant was discarded and the cells were washed with 0.1 M PBS for three times; then, 200 μL superoxide detection solution was added into each well at room temperature for 10 min; finally, the absorbance of each well was measured using a microplate reader (Spectra Max M5; Molecular Devices USA) at 450 nm.

### Quantitative real-time PCR analysis

Total RNA of ADSCs was isolated using TRIzol reagent (TransGen, Beijing, China). Afterwards, the mRNA was reversely transcribed to cDNA by using a Transcriptor First Strand cDNA Synthesis kit (Roche Applied Science, Mannheim, Germany) following the manufacturer’s protocol. The quantitative real-time PCR analysis was performed in an ABI step one plus real-time PCR system (Carlsbad, CA, USA) with cycling conditions as follows: 40 cycles of 95 °C for 15 s, 60 °C for 30 s, and 70 °C for 30 s. The q-PCR primers were as follows: P16 forward primer, CATCTGGAGCAGCATGGAGT; P16 reverse primer, GCCGGATTTAGCTCTGCTCT; P21 forward primer, AGGCACCATGTCCAATCCTG; P21 reverse primer, CTGACCCACAGCAGAAGAGG; P53 forward primer, ATTCAGGCCCTCATCCTCCT; P53 reverse primer, CTCCGTCATGTGCTGTGACT; CXCR4 forward primer, ATCTCCATCACAGAGGCCCT; CXCR4 reverse primer, CAGCTGAGGATCACGGCTAG; β-actin forward primer, CTGGTCGTACCACAGGCATT; and β-actin reverse primer, TGCTAGGAGCCAGAGCAGTA. Gene expression was normalized to that of the β-actin gene, and the relative target gene expression was calculated with the 2^−ΔΔCt^ formula.

### Western blot analysis

Cells were lysed in ice-cold RIPA buffer (0.5 M Tris-HCl, 10 mM EDTA, 1.5 M NaCl, 10% NP-40, 2.5% deoxycholic acid, pH = 7.4) supplemented with protease inhibitor cocktail (Roche, USA). Protein quantification was performed by BCA assay kit (TransGen Biotech, Beijing, China), and loaded samples containing equal amounts of protein (40 μg) were separated by 10% SDS-PAGE. Afterwards, the protein transfer onto nitrocellulose membranes (PALL, USA) was performed within transfer buffer (12 mM Tris base, 96 mM glycine, pH 8.3, and 20% methanol). Subsequently, the membranes were blocked in the TBST buffer containing 5% BSA for 2 h, followed by incubating with the antibody of SOX-2, OCT-4 (1:1000 dilution; all from Cell Signalling Technologies, CST, USA), RUNX-2, perilipin A, and SOX-9 (1:1000 dilution, all from BOSTER Biological Technology, Wuhan, China), and β-actin antibody (1:1000 dilution; TransGen, Beijing, China) overnight at 4 °C. After that, the membranes were washed with TBST buffer for three times, and incubated with secondary antibody (anti-rabbit HRP-conjugated; 1:8000 dilution; Santa Cruz Biotechnology) at room temperature for 1 h. Finally, the expression levels were analyzed by enhanced chemiluminescence and visualized by autoradiography.

### Statistical analysis

Quantitative data was expressed as the mean ± standard deviation (SD). All statistical analyses were performed using the GraphPad Prism version 7.0 (GraphPad Software, CA, USA). Statistical analysis among different groups was performed by unpaired Student *t* test, with *p* < 0.05 considered as statistically significant.

## Results

### Antioxidants promote ADSC cell proliferation during long-term in vitro expansion

**Fig. 1:**
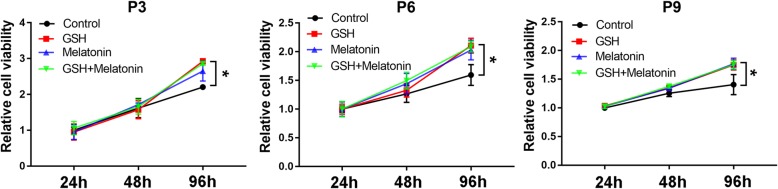
Antioxidants promote ADSC cell proliferation during long-term in vitro expansion. The CCK-8 assay was applied to study the proliferation of ADSCs at passage 3 (P3), passage 6 (P6), and passage 9 after 10 μM GSH or melatonin treatment, or the combination of GSH and melatonin treatment, when the cells were cultured for 24, 48, and 96 h, respectively (*n* = 5 per group; *p < 0.05). ADSCs, adipose tissue-derived stem cells; GSH, reduced glutathione

To determine the effects of antioxidants on cell proliferation during long-term in vitro expansion, different passages of ADSCs (passage 3 (P3), passage 6 (P6), and passage 9 (P9)) were analyzed through CCK-8 assay during in vitro culture. As shown in Fig. 1, the cell proliferation rates of P3, P6, and P9 ADSCs with antioxidant treatment were significantly increased after 96 h of culture compared with the control ADSCs without antioxidant treatment at the same passage, suggesting that the antioxidants could promote ADSC proliferation during long-term in vitro expansion. Interestingly, the combination of GSH and melatonin treatment had no further effect on the proliferation of ADSCs compared with the single treatment, indicating the GSH and melatonin might use the same molecular mechanism to inhibit excessive ROS generation.

### Antioxidants inhibit ADSC cell senescence during long-term in vitro expansion

**Fig. 2:**
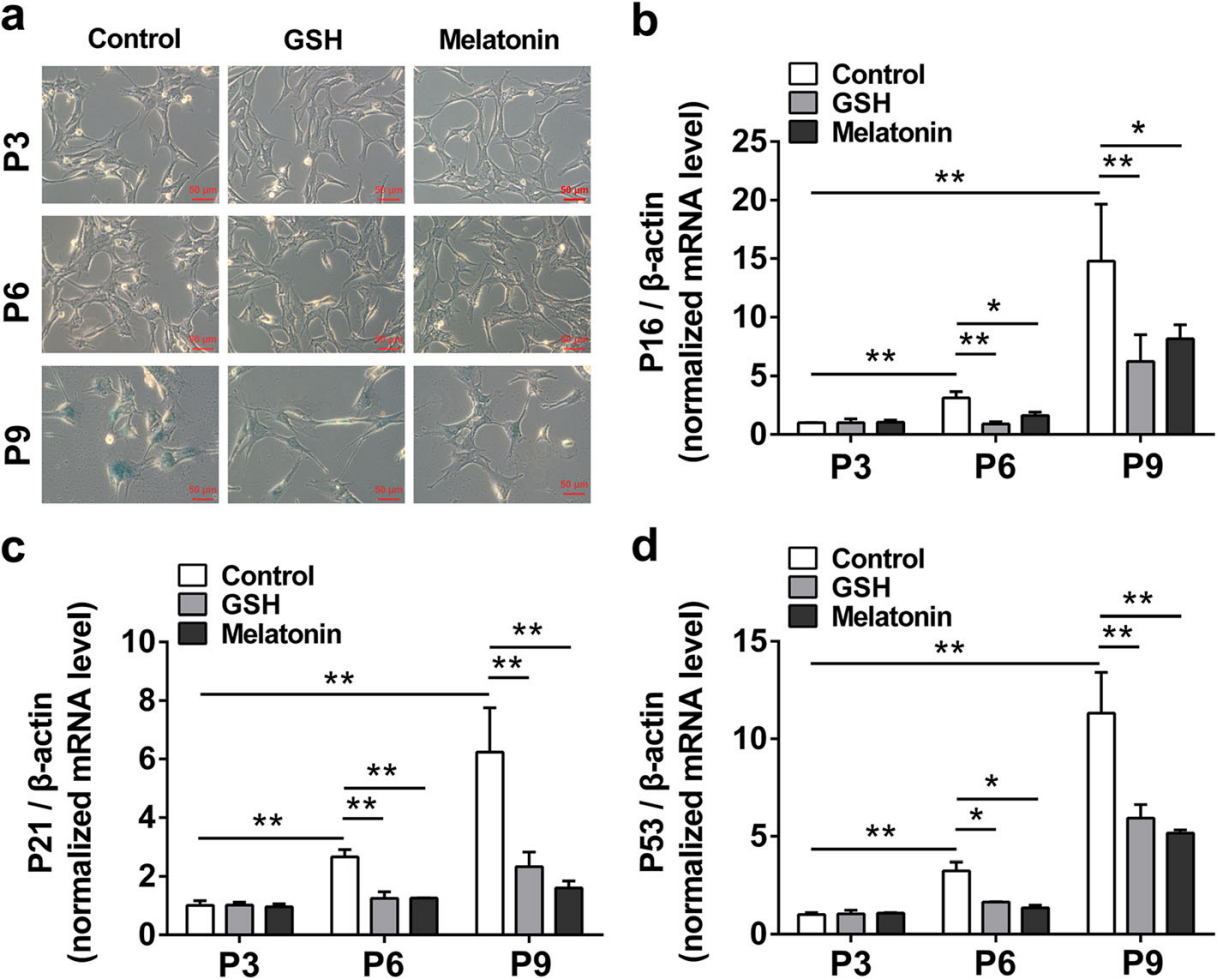
Antioxidants inhibit ADSC cell senescence during long-term in vitro expansion. After treatment with 10 μM GSH or melatonin, the ADSCs cultured for passage 3 (P3), passage 6 (P6), and passage 9 (P9) were used in the following analysis. **a** SA-β-gal staining of ADSCs (× 200 magnification; scale bar, 50 μm). The relative gene expression of p16 (**b**), p21 (**c**), and p53 (**d**) of ADSCs with or without antioxidant treatment in vitro expansion (*n* = 3 per group; *p < 0.05; **p < 0.01). ADSCs, adipose tissue-derived stem cells; GSH, reduced glutathione

To confirm the effects of antioxidants on cell senescence during long-term in vitro expansion, we chose the ADSCs of P3, P6, and P9 for the analysis. As shown in Fig. 2a, there were no senescent cells of P3 and P6 in all groups, while P9 ADSCs showed typical senescence, which means increasing cell aging of ADSCs after long-term passaging; after treatment with antioxidants, GSH and melatonin both reduced cell senescence of P9 ADSCs compared with control P9 ADSCs, suggesting that antioxidants reduced cell senescence of long-term passaged ADSCs; additionally, similar results were also observed using the SPiDER-β Gal fluorescence detection kit (Additional file 1: Figure S1). Given the reducing morphological senescence by antioxidants, we next analyzed the effects of antioxidants on senescence-related gene expression. As shown in Fig. 2b–d, the p16, p21, and p53 mRNA levels were significantly increased in P6 and P9 ADSCs compared with those in P3 ADSCs, which means the undergoing cell senescence of ADSCs in P6 and P9, while these mRNA levels of ADSCs (both in P6 and P9) were decreased after treatment with GSH or melatonin compared with the same passage of ADSCs, suggesting that antioxidants could also inhibit senescent gene expression in long-term passaged ADSCs. Taken together, these data indicate that GSH and melatonin were efficient in reducing cell senescence of ADSCs during long-term in vitro expansion.

### Antioxidants promote ADSC cell migration during long-term in vitro expansion

**Fig. 3:**
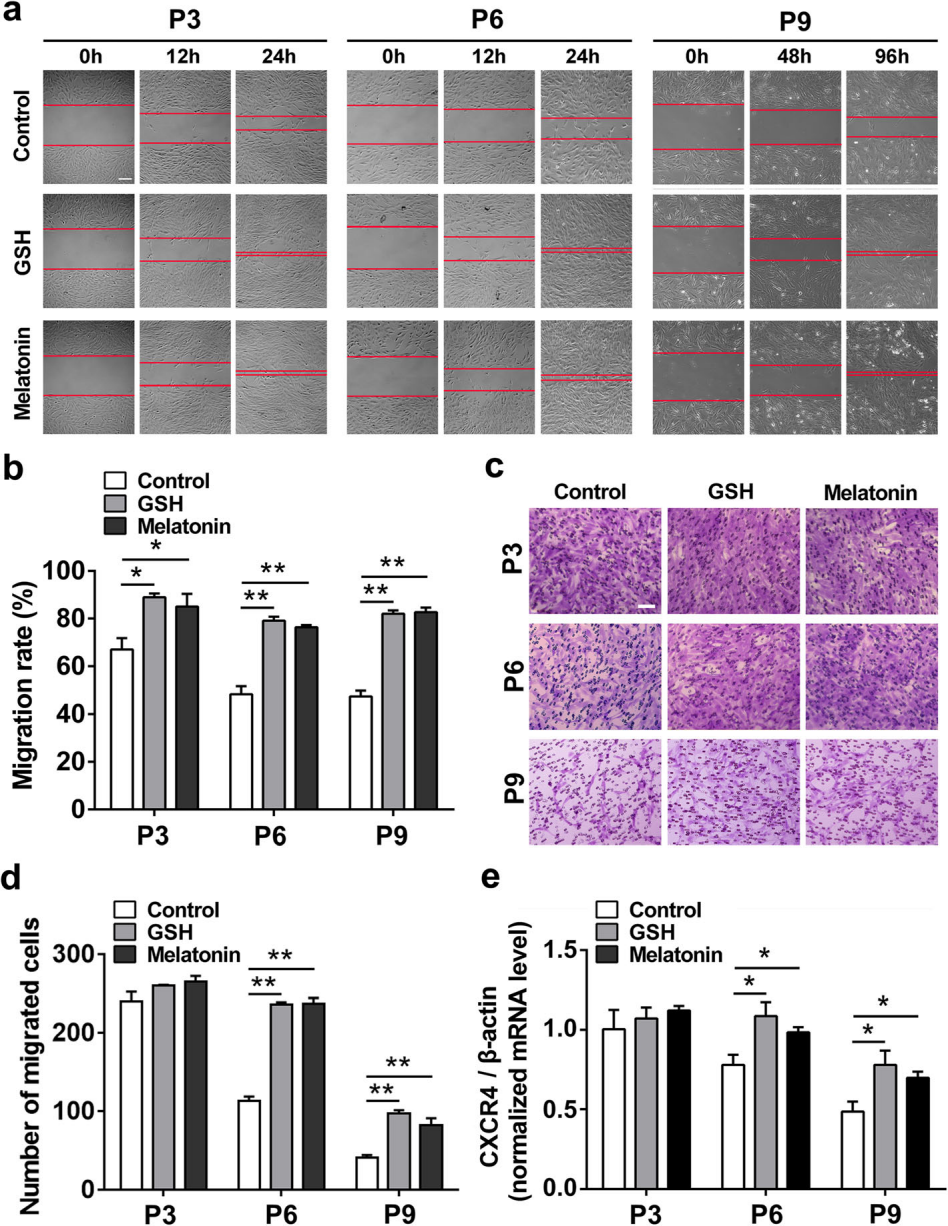
Antioxidants promote ADSC cell migration during long-term in vitro expansion. After treatment with 10 μM GSH or melatonin, the ADSCs cultured for passage 3 (P3), passage 6 (P6), and passage 9 (P9) were used in the following analysis. **a** Motility of ADSCs (× 100 magnification; scale bar, 100 μm). **b** Quantification of the cell motility. **c** Migration of passaged ADSCs (× 200 magnification; scale bar, 50 μm). **d** Quantification of the number of migrated cells (*n* = 5 per group; **p < 0.01). **e** The relative CXCR4 expression of passaged ADSCs with or without antioxidant treatment (*n* = 3 per group; *p < 0.05). ADSCs, adipose tissue-derived stem cells, GSH, reduced glutathione

Considering that cell migrating to the injured sites in vivo is the most important characteristic for stem cell therapy, we next used the wound healing assay to explore the effects of antioxidants on cell motility of passaged ADSCs (Additional files 4 and 5). As shown in Fig. 3a, b, the cell motility was remarkably weakened with the increased passages of ADSCs, which means the impaired cell morality in the progress of in vitro expansion; however, the enhanced motility of ADSCs could be achieved after treatment with GSH or melatonin compared with the same passage of control ADSCs, suggesting that antioxidants could significantly promote ADSC cell motility during the long-term in vitro expansion. Similar to the result of cell motility, the cell migration capability of ADSCs was remarkably decreased during the long-term in vitro expansion, while the enhanced cell migration (in P6 and P9) could be achieved after treatment with antioxidants (including GSH and melatonin) compared with the same passaged control ADSCs, suggesting that antioxidants could promote ADSC cell migration during long-term in vitro expansion (Fig. 3c, d). Since CXCR4 is an important chemokine receptor in regulating stem cell migration and homing [36], we further analyzed the effect of antioxidants on CXCR4 mRNA level of ADSCs. As shown in Fig. 3e, the CXCR4 mRNA expression (in P6 and P9) was significantly increased after treatment with GSH or melatonin as compared with the control ADSCs, implying that antioxidants could promote CXCR4 expression of ADSCs during long-term in vitro expansion. Taken together, these data suggested that antioxidants could significantly promote ADSC cell migration during long-term in vitro expansion.

### Antioxidants preserve stemness and multidirectional differentiation potential of ADSCs during long-term in vitro expansion

**Fig. 4:**
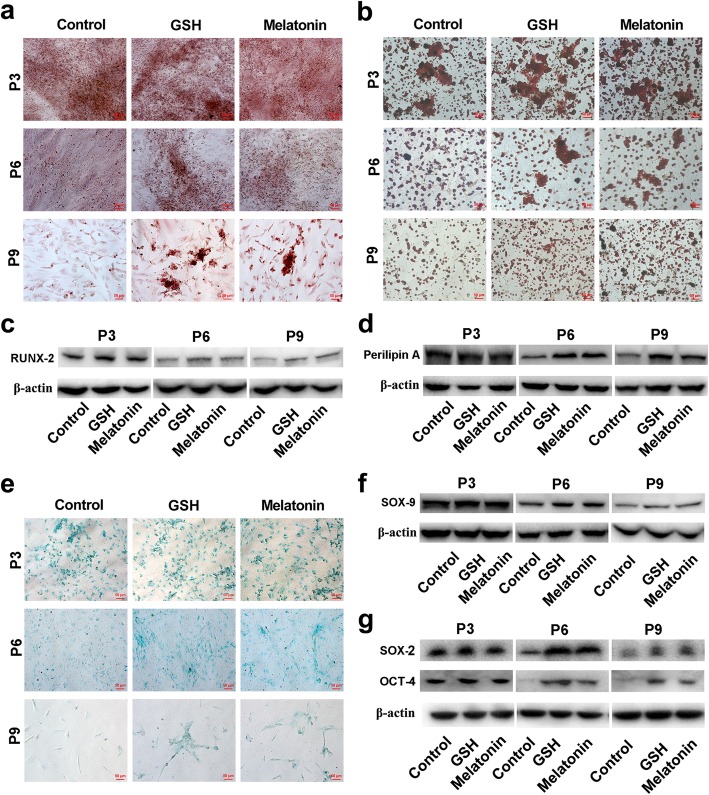
Antioxidants preserve ADSC cell stemness and multidirectional differentiation potential during long-term in vitro expansion. After treatment with 10 μM GSH or melatonin, the ADSCs cultured for passage 3 (P3), passage 6 (P6), and passage 9 (P9) were used in the following analysis. **a** Osteogenesis differentiation of passaged ADSCs (Alizarin Red S staining; scale bar, 50 μm). **b** Adipogenesis differentiation of passaged ADSCs (Oil Red O staining; scale bar, 50 μm). **c** Western blot analysis for RUNX-2 in osteogenic cells. **d** Western blot analysis for perilipin A in adipogenic cells. **e** Chondrogenesis differentiation of passaged ADSCs (Alcian blue staining; scale bar, 50 μm). **f** Western blot analysis for SOX-9 in chondrogenic cells. **g** Western blot analysis for SOX-2, OCT-4, and β-actin in ADSCs. ADSCs, adipose tissue-derived stem cells; GSH, reduced glutathione

Considering that the self-renewal and multidirectional differentiations are the typical characteristics of ADSCs, we next investigated the effects of antioxidants on multidirectional differentiation and stemness of ADSCs. As shown in Fig. 4, the multipotent differentiation ability of ADSCs into adipogenic, osteogenic, and chondrogenic mesodermal lineages, as well as the expression of RUNX-2 (osteoblastic marker), perilipin A (adipocyte marker), SOX-9 (chondral cell marker), SOX-2, and OCT-4, were clearly decreased in P6 and P9 as compared with those in P3 ADSCs, which means the multidirectional differentiation ability and stemness of ADSCs were impaired during the long-term expansion; however, the impaired multidirectional differentiation and the decreased expression of SOX-2 and OCT-4 were reversed after treatment with antioxidants compared with those in control ADSCs, suggesting that antioxidants could preserve the ADSC cell multidirectional differentiation and stemness during long-term in vitro expansion.

### Antioxidants inhibit ROS generation of passaged ADSCs

**Fig. 5:**
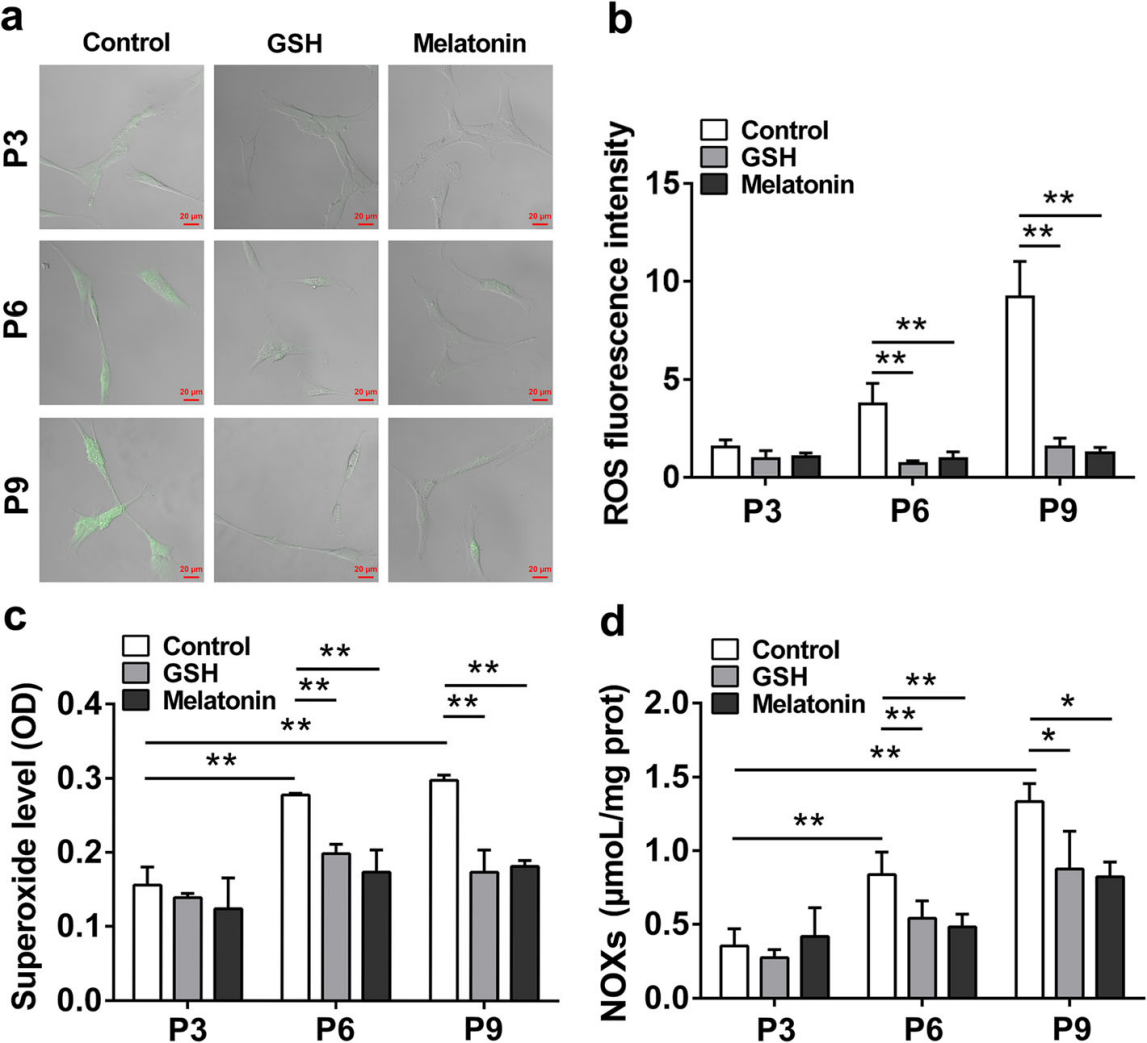
Antioxidants inhibit ROS generation of ADSCs during long-term in vitro expansion. After treatment with 10 μM GSH or melatonin, the ADSCs cultured for passage 3 (P3), passage 6 (P6), and passage 9 (P9) were used in the following analysis. **a** Confocal images of ROS content in ADSCs by DCFH-DA labeling (× 400 magnification; scale bar, 50 μm). **b** Quantification of ROS fluorescence intensity (*n* = 3 per group; **p < 0.01). **c** Superoxide level in ADSCs (*n* = 3 per group; **p < 0.01). **d** NOXs content of ADSCs (*n* = 3 per group; *p < 0.05; **p < 0.01). ADSCs, adipose tissue-derived stem cells; GSH, reduced glutathione; ROS, reactive oxygen species

The above data have shown the protective effects of antioxidants on cell viability and migration, as well as the multidirectional differentiation and stemness of ADSCs during the long-term in vitro expansion. The underlying mechanisms of antioxidants on the reduction of the cell senescence and preserving stemness of ADSCs, ROS, and superoxide in passaged ADSCs were further analyzed since cellular redox homeostasis played a central role in maintaining the stemness and reducing cell aging for stem cells [6, 18]. As shown in Fig. 5a–c, there were no significant differences in ROS and superoxide levels in P3 ADSCs of all groups, while remarkably increased levels of ROS and superoxide were observed in long-term passed ADSCs (in P6 and P9) compared with those in the same passage of control ADSCs, indicating that oxidative stress might occur during long-term in vitro expansion of ADSCs; significantly, after treatment with antioxidants, the decreased levels of ROS and superoxide could be achieved when compared with those in control passaged ADSCs (in P6 and P9), suggesting that antioxidants could inhibit the ROS generation in long-term in vitro expansion. Considering NADPH oxidase (NOXs) as a major source of intracellular ROS generation, the NOXs content of ADSCs was further measured. As shown in Fig. 5d, corresponding with the result of ROS content in ADSCs, the NOXs content was not significantly different in all groups of P3 ADSCs, while the long-term passaged ADSCs (in P6 and P9) showed higher NOXs content than those in P3 ADSCs; accordingly, GSH and melatonin all inhibited NOXs content in passaged ADSCs of P6 and P9 when compared with those in the same passage of ADSCs, implying that antioxidants could inhibit NOXs activity in the long-term expansion. Taken together, these data suggested that antioxidants could reduce ROS generation by inhibiting NOXs activity during long-term in vitro expansion.

## Discussion

Since adipose tissues only had a small proportion of mesenchymal stem cells (MSCs), they must be extensively expanded in culture for ADSC-based therapeutic purpose. However, the lost stemness and cell senescence during in vitro expansion have become a critical issue that hinders the clinical application of ADSC therapy. In the current study, we developed a novel small molecule-based strategy to suppress cell aging and maintain stemness during in vitro expansion of ADSCs. By adding the low dose of antioxidants (including GSH and melatonin) into the in vitro culturing media for ADSCs, we found antioxidants could alleviate cell senescence, promote cell migration, and preserve cell stemness and multidirectional differentiation potential. Importantly, we certified that excessive ROS generation induced by the long-term passaged ADSCs could be effectively inhibited by antioxidants during in vitro expansion. Taken together, our study highlights the promising effect of antioxidants on protecting the cell functions of ADSCs in the long-term expansion.

Increasing evidences suggest that oxidative stress may induce stem cells into premature senescence, causing a series of cellular changes including cell morphological alterations, low cell proliferation, telomere shortening, and reduced genomic stability [37]. In particular, oxidative stress induced by excessive ROS production is the most important factor in stem cell senescence and injuries [38]. Recently, evidence has also been proved that cell aging can be also triggered by a high level of cellular ROS through accelerating telomere shortening [39]. In this study, we found that cell senescence often occurred in the long-term passaged ADSCs (P9) with the high levels of p16, p21, and p53 mRNA. Significantly, we also found the high level of cellular ROS in senescent ADSCs, which suggests that oxidative stress may be an important factor for ADSC aging during in vitro expansion.

Given that stemness maintaining and cell aging are closely related to the oxidative stress induced by ROS [14, 15, 20], it is necessary to reduce the excessive production of ROS during in vitro expansion of ADSCs. In the current study, the low dose of antioxidants including GSH and melatonin was used to inhibit the excessive ROS generation. As predicted, the melatonin and GSH not only had the ability to suppress the high level of ROS in long-term passaged ADSCs, but also could effectively preserve ADSC stemness and reduce cell senescence in long-term in vitro expansion, which may provide a novel approach to preserve cell functions for ADSC expansion.

Endogenous ROS is produced by enzymes including mitochondrial enzymes of the respiratory chain and the NADPH oxidases (NOXs). Among them, NOXs are recognized as the main source of ROS in cells [40]. They catalyze the transfer of electrons from NADPH to molecular oxygen, through the NOX catalytic subunit, to produce ROS, and finally as second messengers regulating cell signaling [41]. In this study, we found that the content of NOXs was significantly increased in ADSCs with high endogenous ROS and superoxide contents, which means that NOX is the major source of ROS generation in the senescent ADSCs. Importantly, after treatment of GSH and melatonin, the NOX content was effectively inhibited with the reduction of ROS generation in ADSCs, suggesting that the antioxidants inhibited ROS generation might partly through suppressing NOXs activities.

Although we showed that antioxidants such as GSH or melatonin could preserve stemness and inhibit cell senescence in mice ADSCs, and similar effects of these antioxidants have also been confirmed on human ADSCs (Additional file 2: Figure S2 and Additional file 3: Figure S3), further studies are still essential to translate this conclusion into clinical usage.

## Conclusion

In summary, we have demonstrated that GSH and melatonin could reduce cell senescence and preserve stem cell functions including cell migration, stemness, and multidirectional differentiation potential through reducing ROS generation in vitro expansion. Therefore, antioxidants may provide a novel strategy for preserving cell functions in ADSC-based therapy.

## Supplementary information

**Additional file 1:** **Figure S1.** Antioxidants inhibit ADSC cell senescence during long-term in vitro expansion. After treatment with 10 μM GSH or melatonin, the ADSCs cultured for passage 3 (P3), passage 6 (P6) and passage 9 (P9) were used in the following analysis. **(a)** Confocal images of SPiDER-βGal staining in ADSCs (×400 magnification; scale bar, 50 μm). **(b)** Quantification of SPiDER-βGal fluorescence intensity (*n* = 3 per group; **p < 0.01). ***ADSCs*** adipose tissue-derived stem cells, ***GSH*** reduced glutathione. (TIF 1019 kb)

**Additional file 2:** **Figure S2.** Antioxidants promote human ADSC cell proliferation. After treatment with 10 μM GSH or melatonin or the combination of GSH and melatonin, human ADSCs from passage 3 (P3) and passage 6 (P6) were respectively cultured for 24, 48 and 96 hours, and the proliferation rate was analyzed by CCK-8 assay (*n* = 5 per group; *p < 0.05; **p < 0.01). ***ADSCs*** adipose tissue-derived stem cells, ***GSH*** reduced glutathione. (TIF 716 kb)

**Additional file 3:** **Figure S3.** Antioxidants promote human ADSC cell migration. After treatment with10 μM GSH or melatonin or the combination of GSH and melatonin, human ADSCs cultured for passage 3 (P3) and passage 6 (P6) were used for migration assay. **(a)** Migration of passaged ADSCs (×200 magnification; scale bar, 50 μm). **(b)** Quantification of the number of migrated cells (n = 5 per group). ***ADSCs*** adipose tissue-derived stem cells, ***GSH*** reduced glutathione. (TIF 3421 kb)

**Additional file 4:** **Figure S4.** Antioxidants promote mouse ADSC cell migration. After treatment with 10 μM GSH or melatonin or the combination of GSH and melatonin, mouse ADSCs cultured for passage 3 (P3), passage 6 (P6) and passage 9 (P9) were used for migration assay. **(a)** Migration of passaged ADSCs (×200 magnification; scale bar, 50 μm). **(b)** Quantification of the number of migrated cells (n = 5 per group). ***ADSCs*** adipose tissue-derived stem cells, ***GSH*** reduced glutathione. (TIF 5004 kb)

**Additional file 5.** Supplementary Material (DOC 33 kb)

## Data Availability

The datasets supporting the results of this article are included within the article.

## Abbreviations

ADSCs: Adipose tissue-derived stem cells
ROS: Reactive oxygen species
GSH: Reduced glutathione
CXCR-4: C-X-C chemokine receptor type 4
SOX-2: Sex-determining region Y-box 2
SOX-9: Sex-determining region Y-box 9
RUNX2: Runt-related transcription factor 2
OCT-4: Octamer-binding transcription factor 4
NOXs: NADPH oxidase
