# Supplementary figures and images for: Antioxidants inhibit cell senescence and preserve stemness of adipose tissue-derived stem cells by reducing ROS generation during long-term in vitro expansion
Source: Stem Cell Res Ther. 2019 Oct 17;10:306. doi: 10.1186/s13287-019-1404-9 (PMC6798439; doi:10.1186/s13287-019-1404-9)

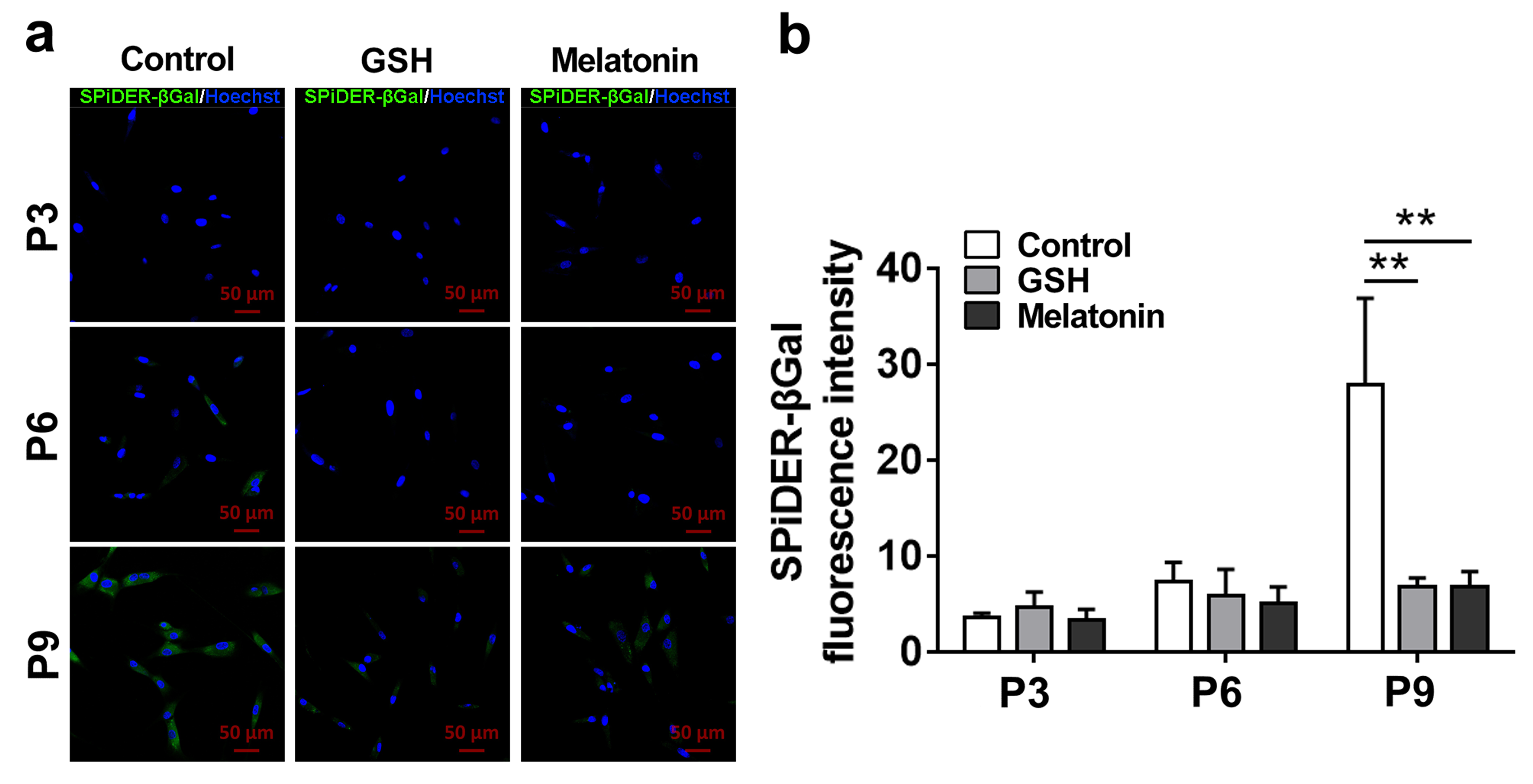

Supplement: Supplementary file 1 — Additional file 1: Figure S1. Antioxidants inhibit ADSC cell senescence during long-term in vitro expansion. After treatment with 10 μM GSH or melatonin, the ADSCs cultured for passage 3 (P3), passage 6 (P6) and passage 9 (P9) were used in the following analysis. (a) Confocal images of SPiDER-βGal staining in ADSCs (×400 magnification; scale bar, 50 μm). (b) Quantification of SPiDER-βGal fluorescence intensity (n = 3 per group; **p < 0.01). ADSCs adipose tissue-derived stem cells, GSH reduced glutathione. (TIF 1019 kb) [file 13287_2019_1404_MOESM1_ESM.tif]

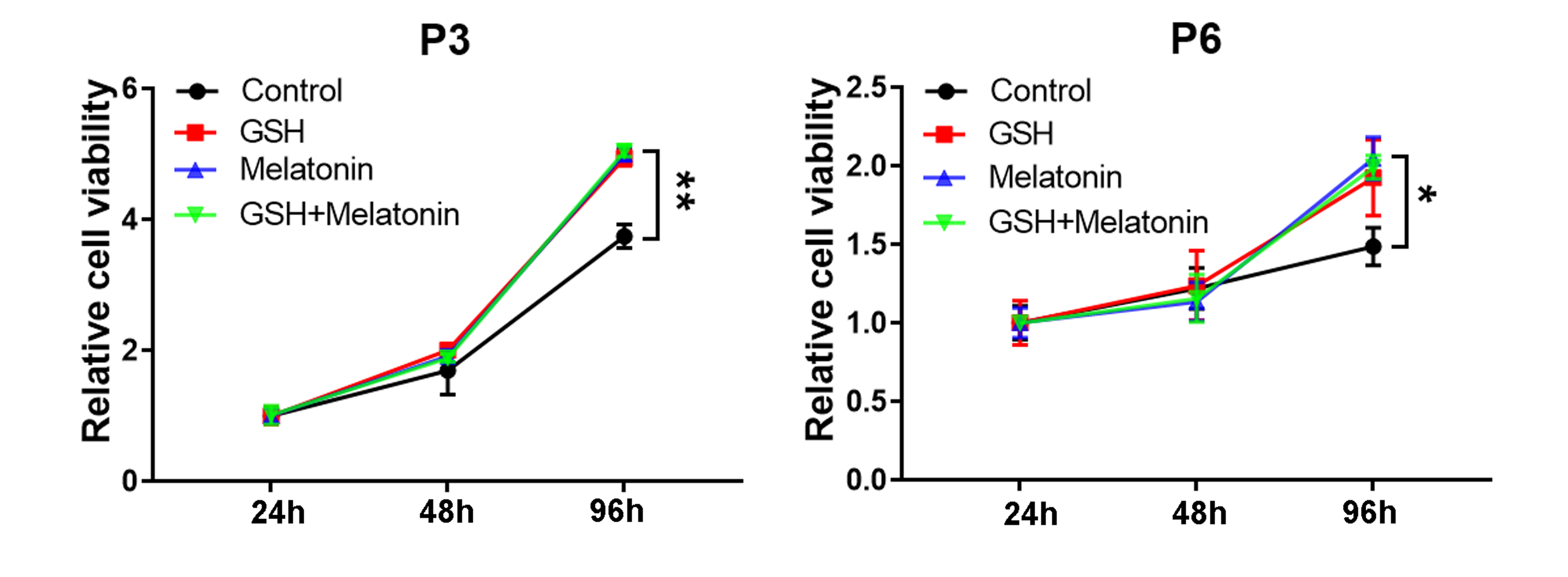

Supplement: Supplementary file 2 — Additional file 2: Figure S2. Antioxidants promote human ADSC cell proliferation. After treatment with 10 μM GSH or melatonin or the combination of GSH and melatonin, human ADSCs from passage 3 (P3) and passage 6 (P6) were respectively cultured for 24, 48 and 96 hours, and the proliferation rate was analyzed by CCK-8 assay (n = 5 per group; *p < 0.05; **p < 0.01). ADSCs adipose tissue-derived stem cells, GSH reduced glutathione. (TIF 716 kb) [file 13287_2019_1404_MOESM2_ESM.tif]

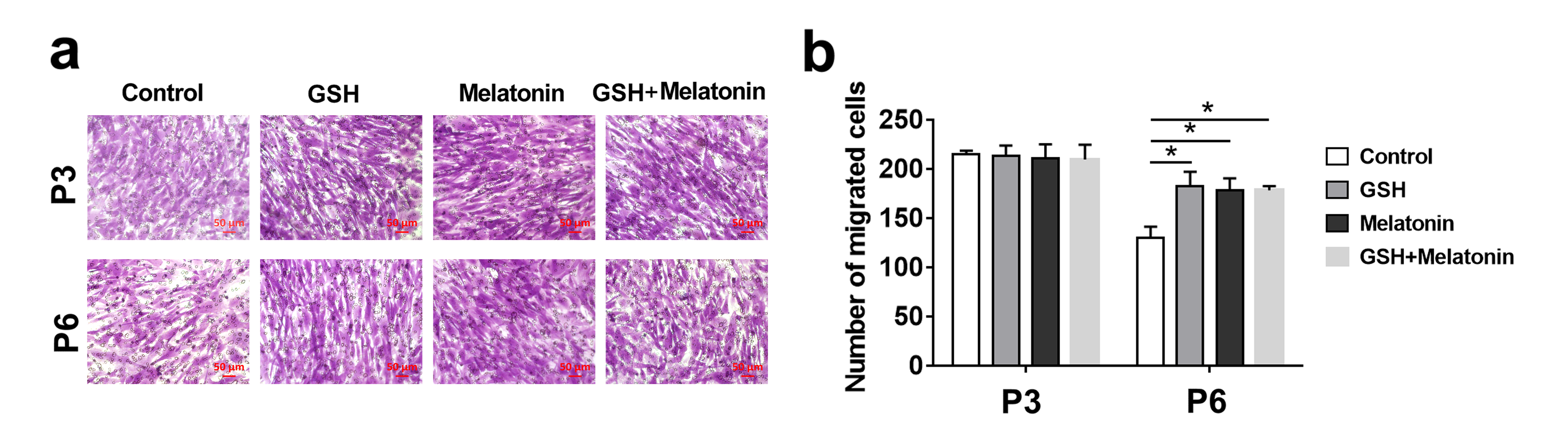

Supplement: Supplementary file 3 — Additional file 3: Figure S3. Antioxidants promote human ADSC cell migration. After treatment with10 μM GSH or melatonin or the combination of GSH and melatonin, human ADSCs cultured for passage 3 (P3) and passage 6 (P6) were used for migration assay. (a) Migration of passaged ADSCs (×200 magnification; scale bar, 50 μm). (b) Quantification of the number of migrated cells (n = 5 per group). ADSCs adipose tissue-derived stem cells, GSH reduced glutathione. (TIF 3421 kb) [file 13287_2019_1404_MOESM3_ESM.tif]

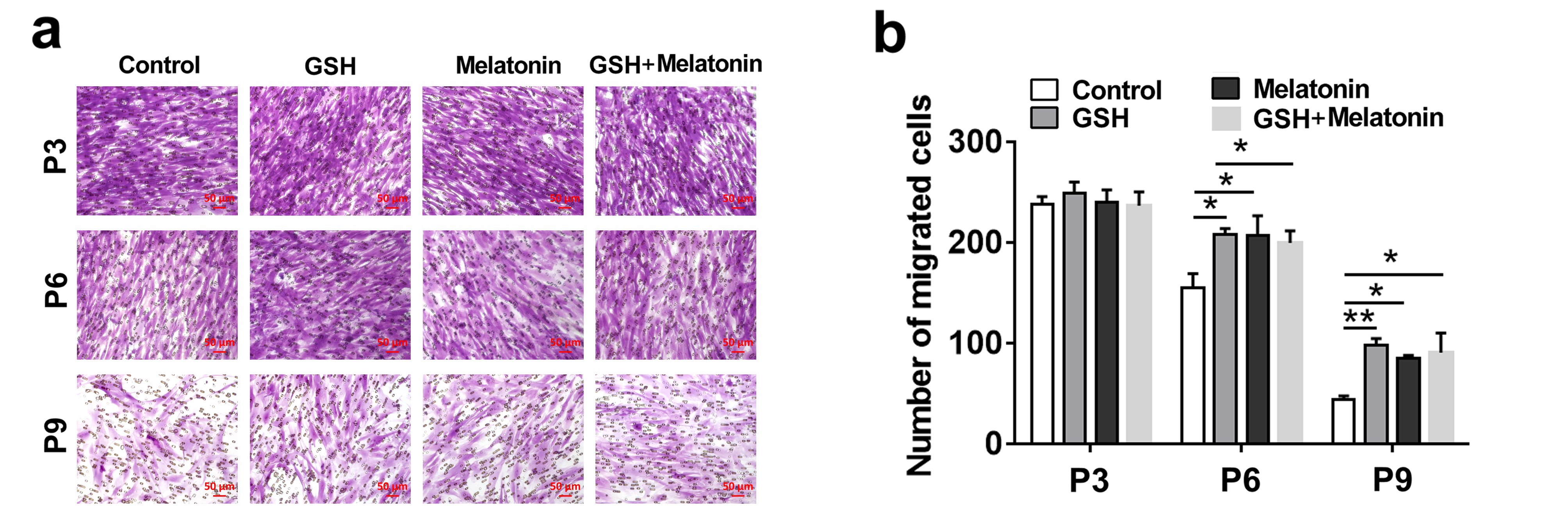

Supplement: Supplementary file 4 — Additional file 4: Figure S4. Antioxidants promote mouse ADSC cell migration. After treatment with 10 μM GSH or melatonin or the combination of GSH and melatonin, mouse ADSCs cultured for passage 3 (P3), passage 6 (P6) and passage 9 (P9) were used for migration assay. (a) Migration of passaged ADSCs (×200 magnification; scale bar, 50 μm). (b) Quantification of the number of migrated cells (n = 5 per group). ADSCs adipose tissue-derived stem cells, GSH reduced glutathione. (TIF 5004 kb) [file 13287_2019_1404_MOESM4_ESM.tif]
