# Supplementary material for: Antioxidants inhibit cell senescence and preserve stemness of adipose tissue-derived stem cells by reducing ROS generation during long-term in vitro expansion
Source: Stem Cell Res Ther. 2019 Oct 17;10:306. doi: 10.1186/s13287-019-1404-9 (PMC6798439; doi:10.1186/s13287-019-1404-9)
Supplement: Supplementary file 5 — Additional file 5. Supplementary Material (DOC 33 kb) [file 13287_2019_1404_MOESM5_ESM.doc]

**Antioxidants inhibit cell senescence and preserve stemness of adipose tissue-derived stem cells by reducing ROS generation during long-term *in vitro* expansion**

Naishun Liao ^1,2,3^, Yingjun Shi ^1,2,3^, Cuilin Zhang ^1,2,3^, Youshi Zheng ^1,2,3^, Yingchao Wang ^1,2,3^, Bixing Zhao ^1,2,3^, Yongyi Zeng ^1,2,3,4^, Xiaolong Liu ^1,2,3*^ and Jingfeng Liu ^1,2,3,4*^

^1^ The United Innovation of Mengchao Hepatobiliary Technology Key Laboratory of Fujian Province, Mengchao Hepatobiliary Hospital of Fujian Medical University, Fuzhou 350025, P.R. China

^2^ Liver Disease Center, The First Affiliated Hospital of Fujian Medical University, Fuzhou 350007, P.R. China

^3^ Mengchao Med-X Center, Fuzhou University, Fuzhou 350116, P.R. China

^4^ The Liver Center of Fujian Province, Fujian Medical University, Fuzhou 350025, P.R. China

**Corresponding author, Correspondence should be address to*: Prof Xiaolong Liu and Prof Jingfeng Liu. E-mail: xiaoloong.liu@gmail.com and drjingfeng@126.com.

**Supplementary Material**

**SPiDER-β Gal staining**

After treating mice ADSCs with GSH and melatonin for passage 3, 6 and 9, the cells were seeded at a density of at a density of 1🞨10^5^/ well in the 35-mm-diameter confocal dishes. 24 hours later, cell senescence was detected by a SPiDER-β Gal staining kit (Dojindo Molecular Technologies, Tokyo, Japan), according to the manufacturer’s instructions. Briefly, the cells were incubated with SPiDER-β Gal for 30 minutes at 37 °C in 5% CO_2_. Afterwards, the cells was washed with 0.01M PBS for three times, and subsequently stained with 10 μg/L Hoechst 33342 solution for 5 minutes at 37 °C in 5% CO_2_. Finally, the stained cells were analyzed by LSM780 confocal microscope (Zeiss, Germany). To further quantify the results, we randomly selected 5 fields (at a magnification of 400🞨) to calculate the fluorescence intensity of each condition. The fluorescence intensity of SPiDER-β Gal was quantified in the ‘Histo’ module of the ZEN 2012 Light Edition imaging analysis system (Zeiss, Germany).

**Human ADSC culture and antioxidant treatment**

Human ADSCs were purchased from the Cell Bank of the Chinese Academy of Sciences (Shanghai, China) and the cells were cultured with StemPro® MSC Sfm ctS™ medium (Gibco, Thermo Fisher Scientifc, Inc.). Human ADSCs cultured with serum-free medium supplemented with 10 μM antioxidant (including reduced glutathione and melatonin) since the first passage. The antioxidant treated medium was changed every 3 days. Once the cells reached confluence, they were enzymatically detached using 0.25% trypsin–EDTA solution (Gibco, USA) and passaged at a ratio of 1:3. Human ADSCs from passage 3 (P3) and passage 6 (P6) were used for cell proliferation and migration assays.
